# Clinical Perspectives on the Notion of Presence

**DOI:** 10.3389/fpsyg.2022.783417

**Published:** 2022-02-25

**Authors:** Pascal Malet, Antoine Bioy, Alfonso Santarpia

**Affiliations:** ^1^Laboratoire Psychopathologie et Processus de Changement (LPPC), Université Paris 8 Vincennes – St Denis, Saint-Denis, France; ^2^Aix Marseille Univ, LPCPP, Aix-en-Provence, France; ^3^Department of Psychology, Université de Sherbrooke, Sherbrooke, QC, Canada

**Keywords:** presence, psychotherapy, embodiment, experiential/existential/humanistic psychotherapy, therapeutic relationship

## Abstract

This article explores the theme of presence of the psychotherapist, a concept that has been of particular interest in humanistic and existential approaches. Presence was first associated with the humanistic attitudes of the practitioner and the way he or she embodies these attitudes in the here and now of the encounter. Since the publication in 2002 of Geller and Greenberg’s model of therapeutic presence, several quantitative studies have explored the relationship between the therapist’s perception of presence and other dimensions of the therapeutic process. However, qualitative explorations still seem necessary to account for the complexity of the therapist’s presence and its role in the therapeutic process. Centered on the therapist’s perspective, we use an idiographic methodology and refer to lived clinical experience to highlight the dimension of sensory contact that, through the body, actualize a connection to a virtual space of the therapeutic relationship. We so describe how a therapist can achieve an embodied processing to clinical material from what we describe as “traces of presence” of the other. From this point of view, the patient’s presence incorporates itself into the therapist’s experience and the therapist can perceive aspects of this presence in a tangible, concrete, and useful way. The therapist’s presence thus takes on a meaning that is not reduced to what the patient will perceive and interpret of his or her attitude. It becomes the main material from which the therapist orients his or her clinical interventions.

## Introduction

Presence may be considered as a posture of action of the therapist in the temporal horizon of the immediate. This posture requires leaving a theoretical description of the practitioner's action and amounts to describing (through literal or metaphorical statements) each singular event (e.g., sensations, perceptions, and mental images) in itself. It requires reintroducing a role for the ephemeral and the unpredictable, approaching each seemingly innocuous event for what it proposes in the patient’s and the therapist’s experience.

We also consider a presence that extends to a global configuration of communication, which could also be a communion between the patient and the therapist. The meaning and value of the therapist’s experiences should not be limited to what the patient will interpret in terms of empathy or therapeutic alliance. Some of these experiences are not ordinary in nature and intensity. They could be understood in terms of a continuum that links the patient and the therapist in an extended context that is not limited to the time and space of the sessions.

This perspective implies attentional receptivity and brings back to the dimensions of contact, of incarnation, of the relationship to time, to space and to objects of perception. Thus, the concept of presence questions the role of these dimensions of the relationship regarding therapeutic change.

The concept of presence is mainly promoted in the field of humanistic and existential approaches (Santarpia, 2020). These approaches achieved a major epistemological shift by conceiving the therapeutic process and its effects in ways that differ from theories of transference and countertransference or of elaboration of the patient’s history. In the therapeutic processes of reproduction, recognition, and repair (Delisle and Mercier, 2010; Santarpia, 2020), which characterize contemporary humanistic approaches, the state of presence activates and fosters the real relationship, which represents the part of the relational process that is established between the therapist and the healthy part of the client. It will therefore be the primary path that restorative acts will take (May, 1967; Delisle, 2004; Bioy and Bachelart, 2010; Santarpia, 2020).

Several studies (Elliott, 2002; Drouin, 2008; Zech, 2008; Elliott et al., 2013; Angus et al., 2015) confirm the effectiveness of humanistic and existential approaches. Meta-analysis by Elliott et al. (2013) and Angus et al. (2015) concluded that, in comparative studies with random groups, clients in humanistic psychotherapy experience change at levels that are as elevated as those of clients undergoing other forms of therapy. So, as other approaches, humanistic therapy in its various forms is an effective means of helping those in distress, and this includes a wide range of mental disorders: depression, anxiety, adjustment, and interpersonal issues (Elliott, 2002; Drouin, 2008).

Many leading practitioners have discussed and considered the role of presence in therapeutic outcomes. The concept is found in the work of May (1967), Bugental (1976), Hycner (1991), and Erskine (2015), as well, of course, as that of Rogers (1957; also see Baldwin, 2013). Geller and Greenberg (2002) presented an initial model of psychotherapeutic presence that is centered on a humanistic approach. They described presence as a state of sensory receptivity to the present moment. This focus on the “sensory and bodily” (Geller and Greenberg, 2002, p. 78) awareness of the experience of self and other can be included in the idea of emotional awareness (Lane and Schwartz, 1987; Santarpia et al., 2020). Notions of presence have also been supported in other approaches. For example, Roustang (2015, p. 192) stated that “The foundation of the therapist’s function is the intensity of his/her presence.”, as Stern (2010, p. 6) referred to the “‘dynamics’ of the very small events, lasting seconds,” that are “the dynamic forms and dynamic experiences of everyday life. The scale is small, but that is where we live, and it makes up the matrix of experiencing other people and feeling their vitality.”

However, as a concept, Presence remains difficult to handle. Hycner (1991) notes that, in the therapeutic relationship, it does not lend itself to objective definitions or clear descriptions.

A first aspect of presence may be associated with the therapist’s humanistic attitudes, particularly the attitudes of congruence, empathy, and unconditional positive regard proposed by Rogers (1957). The therapist takes an active approach to being present (Geller and Greenberg, 2002) in the time and space of the therapeutic session. The effects of these attitudes can be assessed by measuring the patient’s perception of the therapist’s presence.

However, it is not easy to draw a clear line between the concept of presence and the necessary conditions for therapeutic change proposed by Rogers (1957). As a matter of fact, in Geller and Greenberg’s (2002) model of therapeutic presence, presence sometimes appears as a new condition distinct from Rogers’ conditions, as well as sometimes appearing as an embodiment of those conditions. Furthermore, therapists seem to use the term presence to describe different kinds of experiences. There are those which are lived in the relationship with the patient and which can be explained by concepts like congruence or empathy. These experiences are related to a presence connected to the objective dynamics of the interaction. Beside those, there are experiences that are non-ordinary phenomena, described as extrasensory communication experiences by the use of metaphors. They do not seem to be related to an objective temporality in any obvious way.

This leads us to reflect further on the notion of presence and its clinical perspectives. After having presented the difficulties associated with the study of the concept, we will use an idiographic approach. We will highlight the role of the therapist’s receptivity and the possibility of a clinical embodied perspective carried out from a material made of experiences whose nature appears pluralistic. We will show that it is possible to conceive of another form of presence alongside an in-session presence.

More precisely, by taking up Merleau-Ponty and Waelhens (2013), we will underline the dimension of a sensitive contact which actualizes through the body the relation to a virtual space of the therapeutic relationship just as important as the relation to the physical and actual space of the relationship. Through the experience of this sensitive contact, presence may appear as an invisible encounter between two subjective realities that are partially embodied in the course of the intersubjective relation. It is possible to consider a global congruence between the course of the therapist’s pluralistic experience and the objective evolution of the therapy.

## On the Concept of Presence

### Presence and Contact in the Humanistic and Existential Fields

From early on, Rogers held that contact is a necessary and minimum condition for relationships and change: “Two persons are in psychological contact, or have the minimum essential of a relationship, when each makes a perceived or subceived difference in the experiential field of the other” (Rogers, 1959, p. 207; also see Priels et al., 2006).

In this sense, presence involves being available to oneself and the other in a complex way that is centered on the bodily: we welcome the other into our being in a manner that is palpable, kinesthetic, sensual, physical, emotional, and mental (Bugental, 1976). For Erskine (2015, p. 12), presence is associated with the therapist’s “respect, kindness, compassion, and maintaining contact.” Here, we can feel the inspiration of humanistic philosophers, such as Buber (1970), and his “I-Thou” relation, as well as his idea of reciprocity and the rehumanization of the encounter. Hycner (1991) relates presence to the recognition of mystery and the interpenetration of existence; presence requires a willingness to engage oneself entirely in the encounter with the other.

These are the elements that are possible to connect with Rogers’s conditions of congruence, unconditional positive regard, and empathic understanding (Rogers, 1951, 1961). However, Rogers himself drew a distinction between presence and these conditions. In an interview at the end of his career, he said:

perhaps I have stressed too much the three basic conditions (congruence, unconditional positive regard, and empathic understanding). Perhaps it is something around the edges of those conditions that is really the most important element of therapy—when my self is very clearly, obviously present (Baldwin, 2013, p. 28).

He also added, “I recognize that when I am intensely focused on a client, just my presence seems to be healing, and I think this is probably true of any good therapist” (Baldwin, 2013, p. 28).

Concerning the healing power of “just my presence,” Rogers mentions that he makes use of his self and his responses, but that he does not express every aspect of himself. There is thus a subtractive approach to presence, in which irrelevant aspects of the therapist’s personality and experience are set to the side. Presence is what remains when everything else has been removed. This state can be described in terms of its relation to immediacy and embodiment, and one can also wonder about its relation to altered states of consciousness.

### Paying Attention to the Immediate Past, to What Is Immanent in the Moment

Presence occurs in reference to a specific temporal horizon. May (1967) connects it with the notions of the real relationship and the here and now (also see Bioy and Bachelart, 2010). For Yalom (2002), focusing on the here and now means paying special attention to immediate events. This approach is fundamentally “ahistoric” (Yalom, 2002, p. 46), and it takes the intersubjective relationship and the therapeutic relation, understood as a social microcosm, to be the primary factors of therapeutic change.

James (1912, p. 23–24) had already brought the “instant field of the present”—the actuality of its “*naïf* immediacy”—which precedes whatever we can say of it, into the field of psychology, and had given it a primary role.

In his later writings on the therapeutic process, Stern (initially known for his work in psychoanalysis and child development) included insights from phenomenology and certain currents of embodied cognition. He provides a theory of “present moments,” subjective moments of very short duration (Stern, 2004, 2010). In this temporal horizon, every person can participate intuitively in the experience of others, whether in the form of a “micro-drama” (Stern, 2004, p. 22) or a lived emotion that is significant enough for the moment to be grasped without necessarily being verbalized. For Stern, these moments have an impact on the intersubjective field, changing the relationship and allowing everyone to proceed in different directions: “Changes in psychotherapy (or any relationship) occur by way of these non-linear leaps in the ways-of-being-with-another” (Stern, 2004, p. 22).

Centering himself also in this temporal horizon, Roustang—as Le Pelletier-Beaufond (2019) notes—speaks of the attitude of the practitioner; this attitude is one of waiting and expectation, a state of attention to all possibilities and to solutions that have yet to appear, but which are already contained in the present. He locates the psychotherapeutic relationship in terms of bodies that have been positioned in relation to one another well before conceptual thought occurs (Roustang, 2015). He also presents the concept of “perceptude,” which is a manner of being in the world by means of immediate perception; this is located prior to representation and meaning (Le Pelletier-Beaufond, 2019). Perceptude conceives existence as a silent background against which a figure is distinguished. It is a mode of perception that is unaware of time and space.

### Embodiment

In the infinitesimal of a relation, the therapeutic relationship becomes a space where bodies live, move, and locate themselves in respect to one another. The question of presence mingles with those of the body, the living being, and vitality. Since the body only lives in immediacy, returning to the body also means occupying the immediate.

Roustang (2015, p. 174) holds that therapy is a movement of the body in presence: “The positions of the body are at the beginning; they produce and determine the forms of the relationships among people and, as a result, their state of well-being or malaise.” He sought, in his practice, to find the just position. For the therapist, this begins with occupying space and performing a reduction of the human to the animal, setting language and concepts aside. Bodies perceive and notice; they “think before speaking” (Roustang, 2015, p. 175). Roustang states that such embodied thought occurs before conceptual thought, in both the chronological and the structural sense (Roustang, 2015, p. 181). Bodies perceive the subtle clues in basic movements, tones of voice, and expressions. They know where they stand in relation to one another. Between the patient and the therapist, experience becomes immersion or absorption in the body itself, a bringing together of bodily presences and attitudes that are shared by all human beings.

Roustang’s views are supported by approaches concerned with embodied cognition. These posit that there are forms of cognition that do not need to be represented (Gallagher, 2017). The unit that is studied becomes the “organism-environment” pairing (Gibson, 1979, p. 8), and action emerges from the direct interaction between the brain and the environment, without going through any construction of representation. Action thus constitutes a context for the construction of meaning. For Rosch et al. (1993), cognition arises from experiences lived by a body, and meaning emerges from lived and sensory experience that is rooted in the body.

In terms of relationships, Fuchs (2005, p. 98) takes a phenomenological approach that borrows from the field of embodied cognition, describing how human beings can grasp the experience of others virtually; this process is based on an empathic relationship that is not inferential. He notes that the organism can function in an “as if” structure, where preparing for actions relies on processes of perceiving and modeling that are themselves based on motor processes (Fuchs, 2005, p. 103). Such “as if” structures are involved in interpersonal relationships and are made possible by a form of “mutual incorporation” of one another (Fuchs and Koch, 2014, p. 6).

For these authors, emotions arise from the circuit of interaction between the affective qualities or affordances of the environment and the bodily resonances of a subject. Bodily movements that have been amplified or inhibited become the source of lived emotional experience. The concept of affordance, which was suggested by Gibson in 1966 (Luyat and Regia-Corte, 2009), depends on the idea of static or transformational environmental invariants that are perceived by the organism. This explains how an organism will direct its behavior to suit its perception of the possible actions that are provided by its environment. For Luyat and Regia-Corte (2009, p. 315), the reciprocity of the organism and its environment hinges on the perception of affordances and on the organism’s “effectivities,” which are properties of the organism itself.

In these terms, the practitioner’s presence in the patient’s environment can be understood as proposing affordances, actions, and affective investments. What seems superficially to be trivial has the potential to convey meaning for the patient and can do so from the outset of the relationship: what s/he perceives and feels about the spatial organization of the practitioner’s office; how s/he experiences the first contact over the telephone or the first meeting with the therapist, and the therapist’s behavioral invariants. Practitioners should pay attention to these things, especially since, if we follow Roustang, what appears initially becomes essential, in the first instants when bodies take their places and position themselves in relation to each other.

### Geller and Greenberg’s Model of Therapeutic Presence

The first model of therapeutic presence was provided by Geller and Greenberg, on the basis of qualitative research conducted with seven experienced psychotherapists who had written on this theme. They provide the following definition: “Therapeutic presence involves bringing one’s whole self into the encounter with the client, being completely in the moment on a multiplicity of levels, physically, emotionally, cognitively and spiritually” (Geller and Greenberg, 2002, p. 82–83).

They highlight three domains of presence:

– The therapist’s “preparation for presence,” which “occurs *prior to or at the beginning of a session* as well as *in daily life*” (Geller and Greenberg, 2002, p. 75) and involves making oneself available and receptive to the client’s experience. This domain concerns the therapist’s intentionality, philosophy, and other personal practices that are involved in “actively clearing a space inside by putting away personal concerns” that could damage the encounter with the client (Geller and Greenberg, 2002, p. 77).
– The domain of the process of therapeutic presence emphasizes the receptivity of the therapist. This concerns how therapists “touch and are touched by the essence of the client” (Geller and Greenberg, 2002, p. 78), and involves a form of “altered state of consciousness” that encourages “an extrasensory level of communication” (Geller and Greenberg, 2002, p. 78–79). This “received experience” of the encounter as process “is inwardly attended to” in an active and personal way by the therapist (Geller and Greenberg, 2002, p. 79). The domain also includes congruence, one of Rogers’s conditions, in the dimension of authenticity and transparency (Geller and Greenberg, 2002, p. 79).
– Finally, there is the domain of the therapist’s experience of presence in the session (Geller and Greenberg, 2002, p. 80–82), where presence appears as a form of paradoxical experience. Therapists become immersed in the subjective process of the patients, while maintaining a form of objectivity regarding their position in the therapeutic situation. They can also suspend their theoretical knowledge, calling upon it when there is a need to clarify the intuitive responses that emerge in the relational process.

This model of therapeutic presence presents a major advance in the understanding of the notion of presence and its operationalization, by outlining three dimensions of therapist presence. However, clarifications remain to be made in terms of the boundaries of the concept and its use in psychotherapy.

## The Difficulties Associated With the Study of the Concept of Presence

### Non-specific Terms in the Definition

On the basis of the definition only, the notion of presence can appear difficult to apprehend. The following definition proposed by Geller and Greenberg (2001, p.159) makes use of non-specific terms, such as, one’s whole being, being fully in the moment, grounded and centered position: “Presence involves being fully in the moment and directly encountering all aspects of experience with one’s whole being on a multitude of levels—including physical, emotional, mental and visceral—from a grounded and centered position within oneself. Presence is a quality that can be experienced in many life situations, such as art, watching a sunset, teaching, or in quiet meditation with one’s self.”

In another definition (Geller and Greenberg, 2002, p. 72) the authors introduce the notion of intention and movement by using the expression “bringing one’s whole self into the encounter”. The model of therapeutic presence (Geller and Greenberg, 2002) provides important clarifications on the notion of presence and situates it as a foundation and initial condition for the relational phenomena that occur. The definition chosen, however, does not seem to do justice to the model by risking reducing its meaning to an extension of the Rogerian conditions of empathy and congruence.

### On the Boundaries of the Concept of Presence

There seems to be a logical confusion between the concept of presence and Rogers’ nodal conditions of congruence, empathy, and unconditional acceptance of the patient. Geller and Greenberg’s (2002) interpretation places presence both as a precondition, necessary for Rogers’ three conditions and as an independent process. Presence, however, is also seen as the bodily correspondence of the three nodal conditions. Presence thus appears as a concept that is both independent of and intertwined with the three nodal conditions. How then can we situate presence and these conditions, respectively?

One aspect of this problem may lie in the different possible interpretations of Carl Rogers’ conditions. For Tudor (Tudor et al., 2014), there is a tendency to reduce Rogers’ conditions to the three so-called nodal conditions of congruence, empathic understanding of the client’s world and unconditional acceptance of the client. This tendency leads to a form of simplism. It would consider that the three nodal conditions are of the same nature or category, it would ignore the general context of the theory proposed by Rogers and could lead to errors in the conduct of therapy. The first step would be to consider each Rogers’ conditions in their specific nature. For example, Tudor noted that Rogers himself differentiated between the conditions of empathy and acceptance and that of congruence. While empathy and acceptance should be communicated to the patient, congruence, as a way of being of the therapist, would have to be perceived by the patient only to a minimal degree.

In relation to the notion of presence, it appears essential to reintegrate the forgotten conditions of contact, the patient’s state of incongruence, and the patient’s perception. The condition of contact, for example, seems to be underestimated. Considering presence in relation to the notion of contact allows us to bring back the elements of relation to space and time. It is at this level that presence begins and forms a foundation. Similarly, for example, the condition of empathic understanding of the client’s frame of reference has been simplified to “empathic understanding” and then “empathy.” This may have been Rogers’ wish, however, the notion of frame of reference contains the idea of orientation to the patient’s reality, opens up more perspectives, and suggests that the therapist can perceive and feel the way the patient is orienting himself in his life.

How to get out of the logical confusion between presence and Rogerian conditions? A first element may come from Schmid (2002) when he considers nodal conditions as a phenomenological description of presence. But this may be a phenomenological description from the point of view of an observer outside the relationship, or from the point of view of the client. Schmid, however, remains in a position that maintains a confusion between presence and nodal conditions when he states: “Presence is the proper term for the “core conditions” in their interconnectedness as the way of being and acting of the therapist” (Schmid, 2002, p. 81–82). The author speaks of interaction in immediacy but does not make a clear distinction between presence, interaction, and communication. He does, however, mention the link between presence and contact, which supports our idea of reintroducing Rogers’ other conditions for thinking about presence. “In the ‘way of being with’ called ‘presence’ the relationship becomes realisation, and realisation becomes relational: in a certain sense ‘contact’ and ‘perception’ united” (Schmid, 2002, p. 84).

It seems delicate to insert the notion of presence into a pre-existing theoretical system without reorganizing this system in a consequent way. However, nodal conditions may appear to many practitioners as a foundation that cannot be questioned or manipulated. The notion of presence could remain a floating, ill-defined object, even though many practitioners refer to it when talking about their practice. Beyond simply reintroducing the notion of presence in Rogers’ set of conditions, we may then have to explore the relationship between the notion of presence and Rogers’ theory as a whole. For example, Tudor et al. (2014) reminded us that Rogers’ conditions alone do not constitute the center of his theory. It would be to also consider the actualizing tendency of human beings, the principles of non-directiveness, and concept of self. Such a position would lead us to consider presence as a trans-theoretical notion whose contours have yet to be determined and then to observe how presence is embodied in this or that theoretical current.

### Evaluation of the Patient’s Perception of the Therapist’s Presence and Qualitative Discovering of the Therapist’s Presence

Since Geller and Greenberg’s model of therapeutic presence, several quantitative studies have been conducted in an attempt to measure the perception of the therapist’s presence and its impact on other dimensions of the therapeutic process.

Quantitative studies on the therapist’s presence focus on the idea that the patient can evaluate the therapist as more or less present during the session, just as the therapist can evaluate himself or herself as more or less present. This orientation is consistent with one of the six conditions set forth by Rogers (1957), considering the need for the patient to perceive something of the therapist’s attitude toward him or her.

Geller et al. (2010) designed two instruments for measuring the perception of the therapist’s presence. The authors justified the need for such instruments by the fact that there is little research for recommendations that would guide practitioners’ training and practice on the topic of presence. The Therapeutic Presence Inventory (TPI) is available in two versions, therapist (TPI-T) and client (TPI-C). Their validity and reliability were verified by the authors in a study including 25 therapists and 114 clients. The TPI-T contains 21 items allowing therapists to rate their predominant experience of presence/absence on a seven-point Likert scale. The TPI-C contains 3 items allowing clients to rate their therapist’s presence/absence on a 7-point Likert scale.

Quantitative research on presence has often involved measuring perceived presence via the TPI-T and TPI-C and looking for correlations with ratings of the therapeutic alliance, of relationship and empathy, and patient perceived therapeutic change.

A first set of results focuses on the relationship between perceived presence, therapeutic alliance, and therapeutic change. Geller et al. (2010) found that clients’ evaluations of therapists’ presence were associated with their perception of therapeutic change and the quality of the relationship, whereas therapists’ evaluations of their own presence were not correlated with clients’ evaluations of the therapeutic outcome or their perceptions of the relationship. The authors explained this result by the fact that therapists’ evaluations of their own presence would be related to their own experiences of the therapeutic relationship. Therapists would experience presence within themselves without necessarily communicating or expressing it to the client. This result was confirmed by Vinca (2009) who evaluated the therapeutic process of a patient and a therapist over 8 sessions. A client’s perception of his therapist’s presence was correlated with his perception of the quality of the session. However, Dunn et al. (2013) introduced the assessment of the effect of a task performed by the therapist prior to a therapy session. Their research included 25 therapists in their final training, 89 patients, for 132 therapy sessions. It showed that the therapist’s practice of a centering technique for 5 minutes before a psychotherapy session was correlated with better perception of their presence by the therapists and with better perception of the therapist’s presence by the clients.

Another set of results focuses on the relationship between perceived presence and perceived empathy. Pos et al. (2011, cited by Hayes and Vinca, 2017) evaluated the relationship between presence, empathy, and working alliance. The research included 17 therapists and 52 clients with depressive disorders. Results showed a correlation between therapist empathy and presence as rated by clients. However, empathy and presence appeared differently correlated with the working alliance. In this research, the perception of presence predicted the perception of the working alliance, which is not the case for empathy. Presence and empathy thus appeared to be related but also appeared to be distinct concepts. Furthermore, through research with 42 therapists in training and 88 clients followed for an average of 8 sessions, Hayes and Vinca (2011, cited by Hayes and Vinca, 2017) showed that the therapist’s presence perceived by the client was correlated with the therapist’s empathy perceived by the client. It was also found that the more the therapist rated themselves as present, the more the client rated the therapist as empathetic.

The results reported above tend to show that it is difficult for patients to discriminate the presence of other factors in the therapeutic process, such as empathy, alliance, or perceived change. They encourage our discussion above calling for clarification of the links and differences between the concept of presence and Rogers’ conditions for therapeutic change. This clarification will be even more important as therapists themselves do not conceive of presence in the same way. Indeed, in their 2010 article, Geller et al.’s found that cognitive behavioral therapists rated themselves as less present than experiential and person-centered therapists. This result could be explained by a different conception of “presence” within each theoretical approach. Therapists may not currently represent the notion of presence in the same way and do not attach the same aspects of their experience to it, depending on the theoretical approach in which they are involved.

Quantitative studies, while useful, do not capture the full complexity of the internal process followed by therapists and the relationship between this internal process and the course of the therapeutic relationship. Thus, there is a need to further explore the issue of presence through qualitative studies that will allow us to study presence as a subjective process specific to the therapist that should not be reduced to the patient’s explicit perception. Presence engages the therapist, and he has the measure of it through his bodily, sensory experience. Just as only a subject can describe what he or she experiences in trance (Bioy, 2021), only the therapist can describe what he or she experiences in presence.

Two types of qualitative approaches seem relevant to us in this respect: interpretative phenomenological analysis and idiographic approach.

Interpretative Phenomenological Analysis allows for the exploration of how a person makes sense of their experiences, by attempting to determine the meanings from the language and state of mind of the person who is experiencing it. This type of methodology is suitable for studying individual cases before moving on to generalizing to a set of cases (Pietkiewicz and Smith, 2012). For Antoine and smith (2017), the researcher is personally engaged in the research by seeking to interpret the interviewee’s process. He or she is also there as a stimulant of the participant’s reflexivity, to get the participant to engage in the exploration and interpretation of his/her lived experience. Data collection is carried out through semi-structured interviews. The data analysis presents a structured methodology. These elements have been elaborated by Antoine and Smith (2017) and Smith et al. (2009).

We define « idiographic » a science that seeks some particular event (Toomela, 2009), opposed to the nomothetic approach, science that seeks only general laws. Humanistic psychologists and researchers often use the idiographic approach because they believe that a person’s subjective experience is more important to gain an understanding of humans than a universal generalization (Santarpia, 2022). Indeed humanism (Brown, 1972, p. 103) proposes an idiographic perspective:

– man as a whole, indivisible and immediate, superseding the sum of his part processes;
– man is unique, a “once in a lifetime” happening, irreplicable;
– man *qua* man; man assessed in his own terms; man as the measure of man. Nomothetic approach, on the other hand, declares man as an "object" of study, similar to other objects.

An example of qualitative research on the subject of presence is given by Stange Bernhardt et al. (2021), who studied a dyadic case using the Interpretative Phenomenological Analysis. They showed a phenomenon of congruence between the patient’s experience and those of the therapist. The therapist’s way of being was perceived and experienced by both the patient and the therapist.

However, what makes the presence of the therapist a determining factor in the change experienced by patients? Patients undergo a reorganization of their experiences, and many leading practitioners have noted how important it is for them to have experiences that are novel to them. For this reason, special attention is paid to a particular moment in the course of the relationship, one that is a sort of zero-point, where patients become able to have a conscious awareness of what they really feel, in a sensory and visceral sense, without needing to pass this through the filter of their conceptions. In this view, the presence of the therapist offers a return to a vital dimension of experience. The therapeutic relationship, by its connection to immediacy, can activate aspects of lived experience that are located prior to conceptual thought. The therapeutic frame provides a structure for new experiences as well as the means of orienting how they are organized and the modes by which meaning is given to lived experience.

For Rogers (1961, p. 76), this occurs when a person, at a certain point in therapy, can live what he calls the “experiencing of experience.” Through it, the person’s everyday experience can come to include an undistorted awareness of his/her authentic experience. The security of the relationship with the therapist is what allows the patient to “let himself examine various aspects of his experience as they actually feel to him, as they are apprehended through his sensory and visceral equipment, without distorting them to fit the existing concept of self” (Rogers, 1961, p. 76).

While Rogers held that people have to become able to look at aspects of their lived, sensory experience without reducing them to their self-conceptualizations, Erickson stressed the importance of creating conditions in which the unconscious, which he defines as a natural part of human beings, can come to the forefront. This involves reaching a moment that is required for therapy, one in which the patient shows that s/he has lost all self-consciousness (Vesely, 2014).

Roustang, for his part, describes a movement beyond the conceptual dimension, in which humans return to an animal, and the present becomes the place where the therapeutic dimension really takes place:

“This reduction awakens the existence of the individual human as animal, as a primitive being who temporarily lacks the ability to reason. As a result, the past and the future are forbidden. Anyone who lends him/herself to enacting this withdrawal will only be able to apprehend the present” (Roustang, 2015, p. 43).

These authors converge on the idea that one means of therapeutic change involves reaching a moment in which the conceptual and inferential aspects of patients’ experience move to the background, so that they cease to interfere (or interfere less) with embodied and sensory experience. When the patient is less engaged in conceptual inference, lived experience becomes meaningful in reference to the immediate context, which is structured by the therapist’s presence. In this way, people can distance themselves from their complaints and open themselves to new possibilities of meaning and action that are found in the moment.

### The Nature and the Role of the Therapist’s Responsiveness and Activity

The client’s experience of the therapist’s presence does not explain, by itself, how patients can live experiences outside their usual concept of self. So, beside the client’s perception of the therapist’s presence what is the implication of the therapist’s receptivity in achieving therapeutic change?

From a large review of results, Lecomte et al. (2004, p. 89; Santarpia, 2020) listed attitudes of an efficient therapist:

– “Sensitivity to the patient’s characteristics.
– Flexibility in the choice of interventions.
– competence to intervene without inducing a process of resistance.
– finesse in knowing how to follow the patient’s coping styles.
– ability to build a therapeutic alliance.
– affective sensitivity conducive to a secure attachment.
– receptivity to encourage not only empathic responses but also responses of genuine warm acceptance.
– mastery and appropriate application of techniques adapted to the patient’s needs.”

From this perspective, as an openness to one’s own experience, presence could be seen as what guides the therapist in the choice and direction of his/her attitudes and interventions. Bugental’s (1978 1983 1987 1989) notion of presence includes this idea (Geller and Greenberg, 2002, p. 72). He defined presence in terms of three components: “availability and openness to all aspects of the client’s experience”, “openness to one’s own experience”, and the “capacity to respond” to the client based on that experience. It is with one’s own experience that the therapist can encourage venturing outside the usual conceptions of self.

But if presence is about the therapist’s receptivity and engagement of their self in the here and now, how do they do this? Krug (2009) compared the concept of presence of James Bugental and of Irvin Yalom. One idea emerges from his comparison. If presence is about being in the “here and now,” the “here” for Bugental and the “here” for Yalom differs. For Bugental, the “here” refers to the practitioner’s focus on the patient’s subjective activity. For Yalom, the “here” refers to a focus on the intersubjective space. By extension of this idea, we are faced with the fact that the therapist can orient his or her receptivity in multiple ways and have experiences of all kinds of nature and intensity.

### Another Form of Presence

Presence is often understood on a scale of intensity, in terms of presence/absence. Its usefulness is then thought in terms of congruence, in terms of patient’s perception of the empathy shown by the therapist, or in terms of therapeutic alliance. This amounts to considering a presence perceived by the patient. This is obviously essential. However, we need to go deeper into the description, analysis, and use of the therapist’s experiences and perhaps discover other meanings of those experiences.

Beyond the effects of attunements observable in certain moments of the therapeutic interaction, experiences described as extrasensory communication in Geller and Greenberg’s model suggest another form of presence, which appears to us to be of a different nature. Indeed, the therapists studied by the Geller and Greenberg (2002) used terms, such as “sharing sacred space” (p. 78), “empathic resonance with a place that she wasn’t even expressing” (p. 78), “vessel of information…there’s things sort of, again this is esoteric language, sort of moving through me and connecting to me” (p. 79). Those experiences could relate to the notion of “extraordinary presence” proposed by Hayes and Vinca (2017). The authors considered two types of presence. “*Ordinary presence*” would be a prerequisite for Rogerian conditions. It refers to presence that can be experienced by the patient. “*Extraordinary presence*” refers to a “deep state of connexion to oneself and to a fine source of energy of which one is typically unaware” (Hayes and Vinca, 2017, p. 95). It would be difficult to describe and would involve altered states of consciousness. Hayes and Vinca (2017, p. 96) also consider that extraordinary presence would facilitate the therapeutic process regardless of the therapist’s theoretical orientation: “the quality of extraordinary presence is therapeutic in and of itself.” However, the role of this other form of presence still needs to be conceptualized and highlighted.

## An Idiographic Approach Illustrating a Clinical Use of Dimensions of Presence

Through an idiographic approach taken from a sequence led by one of us, we aim to show some clinical interest of another form of presence that we conceptualized as “traces of presence”. It supposes a form of presence not localized to the time of the interaction in session. This form of presence approached by the plural experience lived by the therapist allows an embodied perspective of the clinical process which can be deployed from the use of a raw material which would be located as close as possible to the real experience. It testifies of a global congruence between the course of the subjective experience of the therapist and the course of the therapy thought as a continuum of experience between the partners of the therapy.

### The Therapist

The therapist is a man in his mid-forties. He works in private practice in Reunion Island as a clinical psychologist and psychotherapist. He has completed five years of training in psychology and psychotherapy, is trained in hypnosis, and has been practicing for five years at the time of this follow-up. Before obtaining the title of clinical psychologist and psychotherapist, he had a first 12-year professional life in the field of management and financial direction of organizations, followed by a 5-year coaching/counseling practice of individuals and organizations. The follow-up mentioned below was carried out at the same time as the completion of a doctorate in psychology on the theme of the therapist’s presence.

### The Patient as Seen by the Therapist

Mrs. Arthur is 35 years old, married, and well established in her professional activity. She had already undergone a previous course of therapy with me, which had considerably reduced the frequency and intensity of night sweats. She has a very good capacity for elaboration and integration around the therapeutic work. I saw her again several months later on the basis of an initial request concerning regular cannabis use. With the lockdown decided in France, linked to the Covid crisis, after a first session in the office in physical presence, we carried out three sessions by videoconference, spaced out two weeks each, followed by three other sessions in the office.

### Therapeutic Approach

For Mrs. Arthur, the therapeutic approach took a form of experiential/person-centered therapy. Some aspects of the hypnotic approach were sometimes used to guide Mrs. Arthur in attentional tasks and focus her attention to aspects of her current experience.

In what follows, the therapist describes his experience of a sequence starting with the first videoconference session.

#### Session 1

The previous night before the video conference session, I woke up at about 4 am with the sneaky and surprising “real” vision and impression of Mrs. Arthur standing at the foot of my bed. At the beginning of the session, which took place around mid-morning, Mrs. Arthur did not bring up the topic of cannabis use which had been discussed during the first session and which was the explicit object of her request. She mentions a recurrent difficulty related to her sleep. She describes lucid dreams in which she is aware of being in a dream. In these dreams she tries to solve problems. She says that in the same dream she repeats the same scene several times in an attempt to find a way out of the problematic situation that is taking place. In the dream, solving the problem would allow her to get out of the dream and she says she experiences the anxiety of not being able to get out of the dream. She states that these dreams occur between 4 and 5 am. She says she is very frequently anxious and exhausted when she wakes up. At this point, I obviously associate the hallucination of her presence at the foot of my bed at 4 am and her description of these lucid dreams between 4 and 5 am. I decide to focus the session on the question of these dreams.

During this session, I use a hypnotic induction where the scenario of a peaceful awakening is evoked and repeated several times in multiple forms. Mrs. Arthur responds perfectly to the hypnotic experience proposed and is calm at the end of the session. However, she expresses skepticism and a touch of disappointment about the effects she can expect from the session. However, she resumes her appointment for a psychotherapy session a fortnight later.

#### Session 2

Mrs. Arthur tells me that she was very surprised by the return of a very peaceful sleep and awakening, the night after session 1. She also specifies that the change has been maintained since. The day after session 2, early in the morning, 6am, I am at home and a particular subjective experience takes place in the form of feelings, diffuse and concrete impressions, associated with the impression of locating Mrs. Arthur spatially. I decide to “pretend” that these residual traces of the other’s presence constitute a real presence and that it is possible to establish a form of “dialogue” with this residual presence using writing. On the level of my subjective experience, it was as if I were exchanging with the residual presence of the person as it appeared to me in my bodily experience, in the physical absence of the patient. From an interpretative point of view, this experience calls upon a material made up of certain elements of the patient’s discourse. But also, and above all it was an experience of a tangible presence with which I could interact fictitiously by giving a form to the experience through writing. The text produced, which I have entitled "letter to your presence" and which is reproduced below, was written in one go, by hand.

#### “Letter to Your Presence”

*"You are back. We see each other for a few sessions. You come to talk about guilt. Then you feel better and decide to get on with your life, as you say.*

*Here you are again. It's a few months later, maybe a year. Something has changed.*

*I can feel all the efforts you make not to show sadness. An impression. I tell myself that you are unhappy. And there are like tears inside me. But I know how to control this feeling, it's a piece of information about you, an event in the course of the session, and I tell myself that it doesn't show that I perceive this. I don't talk to you about it at the moment.*

*You talk about your goal. But it doesn't speak to me. It's flat. What is strong is the sadness, the efforts you make to hide it, or maybe you have come to show me the sadness. Maybe you expect me to see a sadness that you can't name.*

*I see this call. A call for someone to hold you, much like a mother comforts her child, or a father rather. It’s like a call to find enveloping contours. Your energy comes to me, it projects itself. There is your body, and another you that projects itself. There is another you calling for help and coming to me for help. She is standing next to your physical you. You do not hear it, you speak to me. I am talking to you and I hear her. Her message is insistent, repeated several times.*

*The images scroll by. I see you cowering, covering your ears. I also have the impression that everything is too open, that there is no longer a place where you are safe and sheltered from the world for a moment.*

*There is the image that you need someone to wrap you up. It is this presence of yourself outside of yourself in fact that seems to be asking for it. I keep in touch with this presence of yourself that you ignore, while talking with you.*

*You talk about your irritations and annoyances. You say you have to control yourself. I notice that the one who experiences the irritation, the one who feels annoyed, her experience, you don't talk about it. I ask you. You say that it is not done to complain, that others must experience the same things as yourself and that they do not complain, that it is up to you to manage.*

*You are looking for a refuge. It seems to me that you find something here, security perhaps. You can talk about him. I'll give you back the words you use. It's not rape after all, you say, even if you say that you feel assaulted sometimes in the marital bed. You should know better than to say no and you say that you love him.*

*I appreciate all the efforts you are making. It moves me inside. You take so much care of the other person's subjective state. You take care not to disturb the other person, to meet their expectations, to avoid any disappointment or frustration. Then you get exhausted and sometimes it explodes.*

*You seem exhausted. And you say so.*

*This word, boundary, came to me from the start, with the vision of you being unhappy. You say it sometimes, you feel something and you tell the other person about it, and then when the other person has spoken, you don't know how to distinguish your thought from theirs.*

*I would like you to learn to make that distinction between what comes from you and what comes from the other, to re-establish a boundary when you wish. To be in the world is to have an effect on what is around you. You would like not to exert this effect, to make as few waves as possible. You feel the waves that are propelled by others, that push you around and you say you can't complain.*

*You cry of course. You can. I don't remember seeing you cry before. You seem to want to explore this question of boundaries. Not too fast, there is no rush. I know you're afraid of the consequences that come with hearing what's talking inside you. You might have to leave, you might have to say to yourself that you did this for nothing. And that's not possible. You've already done this in response to guilt. To give it up would be to face the guilt and then the guilt of not having faced it.*

*You don't talk about the addiction anymore. You can see that it's not the most important thing right away. Now you discover that here you can risk hearing something that comes from inside you. You manage to approach it. From a distance still. For the moment, just to see that this other you is there, even if you don't know how to look at it yet. There is the beginning of an approach”.*

#### Subsequent Sessions

In the session conducted 15 days after session 2, Mrs. Arthur spontaneously and directly mentioned her relationship with her partner. I had indeed associated certain elements of the text relating to the virtual dialogue with the theme of the couple, which Mrs. Arthur had hitherto addressed only in an informative and laconic manner. I believe that post-session 2 experience brought a practical way to bring to maturity a material related to my perception of Mrs. Arthur's internal states and this allowed me to accompany her in a more efficient and fluid way.

Mrs. Arthur’s follow-up continued for 3 more sessions at my office, with work refocused on her relationship with her partner and her perception of her place in the couple. She expressed the need to allow herself to express her limits and needs more freely.

More than a year after the end of her treatment, Mrs. Arthur tells me that she feels fulfilled in her life as a couple. She announces the birth of her first child and the cessation of her use of Cannabis. She is attributing these changes to the psychotherapy.

## Discussion

### Experience and Process of Presence in These Sessions

The sequence illustrated above testifies a posteriori to the diversity and plurality of the therapist’s experience during the session. Geller and Greenberg’s model evokes the alternation between phases of immersion in the relational process and the therapist’s return to an objective position regarding the relationship with the patient.

Only part of the information available to the therapist is explicit during the session. However, it is possible to develop an awareness of and availability to material that can only be described through the use of metaphor. Several elements of the letter to the presence testify to a composite material made up of images, perceptions, sensations, and interpretations and intuitions. This material may have clinical value, for example the therapist’s perception of a dissociation between the person expressing himself or herself verbally and the person they perceive as seeking help of another kind.

As to the nature of this type of experience for the therapist, we can return to James' idea of immediate experience and the pluralistic dimension of experience: “I have now to say that there is no general stuff of which experience at large is made. There are as many stuffs as there are ‘natures’ in the things experienced. If you ask what any one bit of pure experience is made of, the answer is always the same: “It is made of that, of just what appears, of space, of intensity, of flatness, brownness, heaviness, or what not.”…Experience is only a collective name for all these sensible natures, and save for time and space (and, if you like, for ‘being’) there appears no universal element of which all things are made.” (James, 1912, p. 27).

### Traces of Presence

The post-session 2 “letter to your presence” shows that the experience of the therapist that remains as a trace after or between sessions can be processed and transformed in a way that is useful for the therapeutic process.

The therapist conceptualized this process as follows.

“When I am in contact with these patients during a session, sensory and perceptual experience takes the form of a tangible, palpable material that imposes itself on lived experience; this material can be located either inside or outside the body, and can be worked, “handled,” transformed, and used in the clinical situation. It appears as a compendium of information that is not initially comprehensible in an explicit manner, seeming to belong to the dimension of that which initially evades conceptual thought. However, this compendium can unfurl or unfold over time. An instantaneous lived experience can be unfolded over the course of several minutes or more, through an exchange, and a meaning can then emerge from it. These traces of the presence of the other can sometimes be there without my having any idea of what to do with them. I am not required to do anything with them unless they become insistent, but it is also important not to ignore them or try to pretend that they do not exist.

In a session, they provide the sensation that a dialogue is taking place on several parallel lines or levels. One line of communication is located on the verbal level, while another transpires with another dimension of the person; this dimension appears to be dissociated from his/her physical body, yet it remains perceptible and can even be located spatially in his/her vicinity. This other dimension of the person can appear as a sort of insistence, as if a request were manifest on an implicit level, one that is separate from verbal expression. There is thus a contrast between what the physical person is asking for and what comes from something else. At times, it is as though, while the person is speaking, some invisible “other” is communicating in another way, as if “it” were calling out. Sometimes this other “person” is “on hold,” “keeping watch,” remaining “quiet,” “crying out,” or “coming into contact.”

With patients who elicit this kind of experience, certain residual traces persist after the session.

I conceptualized this idea by evoking the notion of residual traces of the other's presence. The spontaneous method that arose on this occasion was structured and rehearsed. It consists in carrying out a dialogue of the as-if type based on the following rules

– writing as soon as possible, but perhaps not too soon in relation to the session. Within 24 hours or more, or at the moment when this presence is felt sufficiently
– writing in the present tense, without reference to your own history or as little as possible. This favors the evocation and description of perceptions and sensations.
– writing in short sentences. Combined with the present tense, they create a particular dynamic that helps in this process
– writing in “one go”
– writing without referring to the person's first name in order to make less reference to already existing concepts about the person
– writing addressing the residual presence while referring to interpretative aspects of the experience in relation to this residual presence. Describing not only moments, perceptions, sensations, emotions, but also what comes to mind, from what has already been elaborated about the person, from the fiction we have constructed of him/her. There are things that may have been said and also all those things that were not thought or said during the process. It is as if what has been said initiates other things that emerge outside the session
– writing with compassion in mind
– As few contextual elements as possible. The residual presence becomes the only element of our context.
– Like in dreams, there is no anteriority to the scene described. Just what comes in reference to that scene; we describe the material contained in that scene, then another scene is there, etc. The scenes follow one another without necessarily an anteriority or chronology.

In doing this, I was, on one hand, giving a concrete form to the wealth of information that had been incorporated over the course of an interaction. Carrying out an “as if” sort of conversation with the incorporated residual presence allows the available compendium of information pertaining to the therapeutic relationship, which has not yet been put into words, to be actualized. On the other hand, I was able to observe that this way of proceeding has specific effects. For example, following such a “remote session,” the residual presence changes and becomes less distinct. It can also happen that it leaves me with a foreknowledge of the patient, in which certain elements that were located at the margins of what s/he said become central in the written conversation, and then emerge spontaneously in the therapy session, when the patient brings them up as the main theme. Similarly, other elements that were implicit then become explicit for me in my interaction with the residual presence, and later become explicit for the patient. Finally, this work with my lived experience of the other’s residual presence seems to enhance my long-term sensory and perceptual acuity.”

### Virtual Space, Sensory Contact, and Traces of the Presence of the Other

How can we understand this type of experience, which suggests that there is a form of responsive, sensory contact between patient and therapist? Merleau-Ponty’s (1966) early concepts of virtual space, virtual movement, and the virtual body are helpful here.

Merleau-Ponty’s definition of virtual space is based on the work of Wallon in the 1920s. As Parmentier (2018, para. 7) notes, Wallon conceived of virtual space as a place where “nascent or aborted movements,” whose “physical component is repressed…and remains only in the state of a trace,” can appear. Parmentier then adds that, for Merleau-Ponty, virtual space is composed of a “cluster of [possible] trajectories” (para. 17), an extension of the body, which is understood as “the living envelope of our actions” (Merleau-Ponty, 1942, p. 188; cited in Parmentier, 2018, para. 17). In order to perform a movement, a human being would have to be located in virtual space, while also being located in physical space. Whereas physical movement takes place in the actual, abstract movement takes place in the non-actual, that is, the possible (Parmentier, 2018, para. 23).

As a result, the place where the therapeutic relationship occurs can be interpreted as one in which the therapist deploys a presence in terms not only of what happens in the physical space or in his/her thoughts, but also of what is going on in the virtual space. One hypothesis is that an aspect of presence involves the psychotherapist’s ability to grasp and actualize in an embodied manner the virtual space of the relationship. This space contains gestures and movements that are not performed in any actual space. When bodies are in presence, their movements may be “on the way,” interrupted, or held back; they may not find a means of expression in the physical space or they may be repressed. In all of these cases, movements exist as possible, in a non-physical or virtual space. They are not perceived consciously; instead, they are actualized by the psychotherapist as traces in the lived body, without actually being conceptualized. The therapist’s use of these movements—which exist in the possible and not yet in the physical—by the intermediary of his/her bodily experience, may encourage the patient to (re)turn to new possibilities. This use guides the patient’s experience beyond his/her habitual concepts and ways of thinking. In this way, the patient can add to the sum of all his/her experiences, as Rogers (1957) suggests.

Presence thus becomes presence to the virtual space as much as to the physical space or to the already conceptualized aspects of immediate experience. It may also be a sign of the therapist’s relation to a virtual body, a relation that develops over the course of professional practice in his/her relations to patients.

### Presence, *relation*, *Relation*, and Therapeutic Change

Presence thus appears to be intimately linked to the capacity to actualize the information resulting from the relation to the virtual space as well as to the actual space of the relation. It allows the emergence of a clinical material which is elaborated from the presence of the other as it appears in the experience of the therapist. This presupposes the existence of a primary disposition to welcome the other in our psychic reality.

We refer to Buber’s groundbreaking work of 1923, *I and Thou*, which has served as a point of reference for humanistic and existential approaches. For (Buber, 1970, p. 82), the world is twofold for humans, in accordance with our twofold attitude. These attitudes and worlds appear bound to the way in which time and space are considered.

In the first attitude, humans perceive and treat perceived objects as things, whether they are things, facts, actions, or living beings. In this attitude, things are conceived of as situated and included in space and time; they are compared with each other and organized in a world that human beings cannot do without. Buber explains that “although [this world] takes a somewhat different form for everybody, it is prepared to be a common object for you” (Buber, 1970, p. 83). While the “reliability [of this world] preserves you,” it is also true that “you cannot encounter others in it” (Buber, 1970, p. 83).

The relational effects where the patient perceives the therapist’s empathy or acceptance could be located in the temporal dynamics of the therapeutic relationship. They are part of the facts that can be objectified by observing and comparing the discourses between patient and therapist.

It could be different for the phenomena that we have tried to describe concerning the therapist and Mrs. Arthur. Buber describes an other attitude in which human beings do encounter “being and becoming” (Buber, 1970, p. 83). This encounter is unique each time, involving “always only one being and every thing only as a being” (Buber, 1970, p. 83). Further, “The encounters do not order themselves to become a world, but each is for you a sign of the world order. They have no association with each other, but every one guarantees your association with the world” (Buber, 1970, p. 83). The world that appears in this way is “unreliable”'; it lacks both density and duration (Buber, 1970, p. 83). In this attitude, the relation to time and space has been reversed. In the first world of things, the “It-world,” we consider things to exist in a space and a time; in the second world, the “You-world,” space and time are included in each encounter (Buber, 1970, p. 84).

This idea goes hand in hand with Buber's distinction between “*relation*” and “*Relation*.” As *relation* is situated in time and space, *Relation* constitutes a *primary fact*, a *welcoming disposition*, a *psychic mold*, a global configuration which allows the encounter and in which the relationship to time and space will be structured.

In support of these ideas, we can consider therapeutic presence from the point of view of *relation* and the active part by which the therapist engages his or her presence in the encounter with the patient. And we can consider presence from the point of view of *Relation* as a first global disposition of welcome to all that this *psychic mold* can contain and propose in terms of natures and intensities of experiences. Presence here starts as something immanent. Presence in this case appears as a global configuration that is conceptually situated before the physical encounter and that structures the physical encounter.

We can speak of a willingness to let our experience of the other take on its full shape and suspend the usual course of our relationship with the world. In what may appear as altered states of consciousness, space and time are no longer separate elements or the containers of our experience. In the case presented above, for the therapist, a disposition to a stream of experience related to the patient is activated at the time of the appointment. Part of this activation seems to be quite implicit and not located in the time of the session. We understand this as a readiness for the presence of the other in our experience. This activation can result in specific experiences, such as the hallucination of the patient’s presence on the morning.

The relationship to the other and to the world becomes then an intimate experience. It is from within the variation of these states of consciousness that the human and clinical relationship is lived. The therapist perceives the moment when Mrs. Arthur speaks about her lucid dreams as something significant but also normal as he is immerged in the encounter. But as much these events appear meaningful and normal in the moment of the experience, so much they can appear as strange or simple coincidences when seen from the outside.

On another note, moments can be observed in the therapeutic process where there is a duality between what Mrs. Arthur considers to be her problem and what the therapist perceives and uses:

– the patient’s request for support in relation to an addiction evolves into a request concerning her sleep and then her relationship with her partner
– her skepticism at the end of session 1 and her surprise to see the absence of disorders after the session and in a lasting way
– the perception by the therapist of an implicit request different from the one expressed verbally, and the fact that this other request, at first on the margin, will become central later on.

These elements seem to confirm the “need” for the patient to have experiences outside of the usual conceptions of self, as discussed above, and the place of immediate and pluralistic experience in this process.

It is also observed that a continuum linking the patient and the therapist was in motion, remarkable for the mirrored experiences, such as that of the therapist’s hallucination and the patient’s dream, or that of the letter to the presence, which account for a process internal to the patient as perceived by the therapist, at first at the margin in her discourse and becoming central in the later sessions. The therapist’s subjective experience translates or even anticipates the evolution of the relational continuum and clinical process.

### Further Directions

We have attempted to outline some of the clinical perspectives enabled by the concept of therapeutic presence. The therapist’s posture centered on immediate experience and his availability to pluralistic experiences generates a body of information with potential value and role in therapeutic change. The therapist’s availability to immediate experience is as much about an availability to actual as well as virtual aspects of experience. Presence takes shape in a continuum between patient and therapist and gives rhythm to therapeutic change.

Future research should address the confusion of logical levels between presence as a concept and Rogers' attitudes. This confusion of logical levels may come from a confusion between two types of presence. One presence would be associated with the therapist’s posture and the dimensions that he mobilizes and invests in order to get in touch with the patient. Another presence as proposed by Hayes and Vinca (2017), could be qualified of ‘*extraordinary presence*’ and probably belongs to another conceptual category. Should we even distinguish in the concept of presence, three aspects of presence:

– Presence of the therapist as perceived by the patient and which supports the quality of the relationship
– Presence as an embodied perspective characterized by body-related processes including the integration of perceptions and representations and by which the therapist deploys clinical interventions
– Extended or extraordinary presence which testifies to the therapist’s state of deep connexion to oneself and to a global configuration of communication with the patient. This type of experience is difficult to conceptualize, and its description requires the use of metaphors.

In any case, it seems important to deepen the research about presence that guides the therapist and extraordinary presence. Hayes and Vinca (2017) think that it is rare and difficult to find and to measure extraordinary presence. However, this type of presence could be much more frequent than we would think. We believe that therapists can train themselves to be more open and receptive to fine dimensions of their experience that reflect the relationship at a deeper level.

In our opinion, the concept of “traces of presence” could prove to be an interesting concept to guide therapists in the observation of variations in their own altered states of consciousness. It could help to recognize and describe our experiences with greater accuracy to account for their plural nature while considering their value as a guide or as a therapeutic principle.

In this sense, presence of the therapist is primarily his or her receptivity to his or her own experience of the patient’s presence. It represents his ability and willingness to engage his or her sensitivity in an interaction (from interaction to communication or even communion) with the patient’s presence, simultaneously at different dimensions of the relationship, in a way that is experienced as tangible, palpable, and concrete and in a context that is intended to be therapeutic. These different dimensions are in particular:

– Temporal. They imply changes in the orientation of time with a focus on the horizon of the smallest moments, the immediate, the birth of movements.
– Spatial. They imply an orientation of the therapist to the actual or virtual space of the relationship.
– Corporeal. They imply the relation to the actual body and movements as well as to the virtual body and movements, and the relation to the traces of the patient’s presence in the therapist’s bodily experience.
– Identity. They imply putting aside aspects of the self that should not interfere with the encounter.

This presence is inscribed in a general context of the relationship that is not limited to objective time and space. It produces perceptual, sensory, emotional, and conceptual material that the therapist can use to set up the conditions for therapeutic change.

## Data Availability Statement

The raw data supporting the conclusions of this article will be made available by the authors, without undue reservation.

## Ethics Statement

Ethical review and approval were not required for the study on human participants in accordance with the local legislation and institutional requirements. The patients/participants provided their written informed consent to participate in this study.

## Author Contributions

PM: conceptualization, methodology, investigation, and writing original draft. AB and AS: conceptualization, supervision, and writing—review and editing. All authors contributed to the article and approved the submitted version.

## Funding

Université Paris 8 Vincennes – St Denis: contribution for translation from French to English

## Conflict of Interest

The authors declare that the research was conducted in the absence of any commercial or financial relationships that could be construed as a potential conflict of interest.

## Publisher’s Note

All claims expressed in this article are solely those of the authors and do not necessarily represent those of their affiliated organizations, or those of the publisher, the editors and the reviewers. Any product that may be evaluated in this article, or claim that may be made by its manufacturer, is not guaranteed or endorsed by the publisher.
